# A feather star is born: embryonic development and nervous system organization in the crinoid *Antedon mediterranea*

**DOI:** 10.1098/rsob.240115

**Published:** 2024-08-21

**Authors:** Silvia Mercurio, Giacomo Gattoni, Giorgio Scarì, Miriam Ascagni, Benedetta Barzaghi, Maurice R. Elphick, Jenifer C. Croce, Michael Schubert, Elia Benito-Gutiérrez, Roberta Pennati

**Affiliations:** ^1^ Department of Environmental Science and Policy, Università degli Studi di Milano, Milan, Italy; ^2^ Department of Zoology, University of Cambridge, Cambridge, UK; ^3^ Department of Biosciences, Università degli Studi di Milano, Milan, Italy; ^4^ Unitech NOLIMITS, Università degli Studi di Milano, Milan, Italy; ^5^ School of Biological and Behavioural Sciences, Queen Mary University of London, London, UK; ^6^ Laboratoire de Biologie du Développement de Villefranche-sur-Mer (LBDV), Institut de la Mer de Villefranche (IMEV), Sorbonne Université, CNRS, Villefranche-sur-Mer, France; ^7^ Department of Neuroscience, Genentech, South San Francisco, CA, USA

**Keywords:** echinoderm development, nervous system, apical organ, skeletogenesis, crinoids, evolution of development

## Abstract

Crinoids belong to the Echinodermata, marine invertebrates with a highly derived adult pentaradial body plan. As the sister group to all other extant echinoderms, crinoids occupy a key phylogenetic position to explore the evolutionary history of the whole phylum. However, their development remains understudied compared with that of other echinoderms. Therefore, the aim here was to establish the Mediterranean feather star (*Antedon mediterranea*) as an experimental system for developmental biology. We first set up a method for culturing embryos *in vitro* and defined a standardized staging system for this species. We then optimized protocols to characterize the morphological and molecular development of the main structures of the feather star body plan. Focusing on the nervous system, we showed that the larval apical organ includes serotonergic, GABAergic and glutamatergic neurons, which develop within a conserved anterior molecular signature. We described the composition of the early post-metamorphic nervous system and revealed that it has an anterior signature. These results further our knowledge on crinoid development and provide new techniques to investigate feather star embryogenesis. This will pave the way for the inclusion of crinoids in comparative studies addressing the origin of the echinoderm body plan and the evolutionary diversification of deuterostomes.

## 1. Introduction

Echinoderms are some of the most enigmatic animals among deuterostomes, the taxon that includes ambulacrarians (i.e. echinoderms plus hemichordates) and chordates. Echinoderms display a highly derived adult body plan, characterized by a pentaradial symmetry, a calcitic endoskeleton, a coelom-derived water vascular system and a peculiar nervous system, in which the presence of a centralized, integrative centre is still a matter of debate [1,2]. Modern echinoderms are divided into two main taxa: the Eleutherozoa, comprising four extant classes (asteroids, ophiuroids, echinoids and holothuroids), and the Pelmatozoa, of which crinoids are the only living members [3]. The crinoids can further be subdivided into stalked sea lilies, which remain sessile after metamorphosis, and stalkless feather stars, which detach from their stalk after post-larval development and become free-living adults [4,5].

As the sister group to the rest of the echinoderms, crinoids are of key importance for understanding the evolutionary history of the phylum [6]. However, knowledge of crinoid biology and development remains scarce compared with the wealth of information on the life cycle of eleutherozoans. Sea urchins, for example, are traditional models in developmental biology, and their early embryogenesis and larval development have been thoroughly investigated [7,8]. With the advent of molecular biology, eleutherozoans have further been used to study gene regulatory networks and their role in the specification of cell types [9–11]. More recently, optimized culturing protocols for research and commercial purposes have made it possible to follow their development through metamorphosis and to study juvenile stages [12–14]. Conversely, apart from early morphological descriptions [15,16], to date only a few studies have investigated crinoid embryogenesis and metamorphosis [17–20], despite an increasing interest in crinoid biology within the scientific community [4,21,22].

Crinoids have swimming ciliated lecithotrophic larvae, with sea lilies exhibiting an early dipleurula-like stage followed by a barrel-shaped doliolaria and feather stars featuring only a doliolaria stage [17,18,23]. It was shown previously that doliolaria larvae have a nervous system characterized by a diffuse basiepithelial neural plexus and an anterior serotonergic apical organ, located below a tufted apical pit [4,19]. While apical organs are a conserved feature of echinoderm development, the molecular networks leading to their differentiation as well as to that of other larval neural structures have only been dissected in eleutherozoan models [7,11,24]. After a short larval phase, the doliolaria settles on the seabed and undergoes a gradual metamorphosis, during which the larval tissues are rearranged to form a transient cystidean stage that develops into a sessile pentacrinoid [4,17,25]. The majority of recent studies on crinoid development have focused on these post-embryonic stages [5,22,26], while the molecular cues guiding embryogenesis still remain mostly unexplored. This is partly due to difficulties in collecting wild specimens and in manipulating crinoid gametes and embryos.

Crinoids are more abundant in the Indo-Pacific region, where many stalkless crinoids inhabit coastal shallow waters. They are also common in cold waters at depths between 15 and 150 m, while sea lilies are generally regarded as deep sea animals [16,27]. Moreover, crinoid reproductive seasons are extremely variable, being mostly population-specific and cues controlling spawning are largely unknown [4,17,28,29]. *Antedon mediterranea* (electronic supplementary material, figure S1) is a Mediterranean feather star traditionally used for regeneration studies [30,31]. It is an externally brooding animal [32], and embryos are retained on the genital pinnules of the female until they hatch. Early embryos are thus difficult to access and to experimentally manipulate, significantly complicating the use of *A. mediterranea* as a model in developmental and evolutionary biology [4,17]. To overcome this difficulty and to establish *A. mediterranea* as a new model system, we set up a method for culturing embryos *in vitro* from zygote to hatching larva and established a standardized staging system of feather star development based on the main features that we described for each developmental stage. We further optimized whole-mount immunohistochemistry and *in situ* hybridization chain reaction (HCR) protocols to trace the origin and development of the main larval structures. Finally, while focusing on the nervous system, we characterized related cell populations and conserved molecular signatures in larval and post-metamorphic stages. By providing the first analysis of gene co-expression in crinoids, together with the localization of neurotransmitters, we identified a complex apical organ in the feather star larva that develops within a highly conserved anterior domain. Taken together, our results deepen knowledge of crinoid development and provide a set of techniques that will facilitate the use of the crinoid *A. mediterranea* as an experimental model in developmental and evolutionary studies.

## 2. Results

### 2.1. Spawning induction, embryo collection and *in vitro* embryo culture

**Figure 1.**
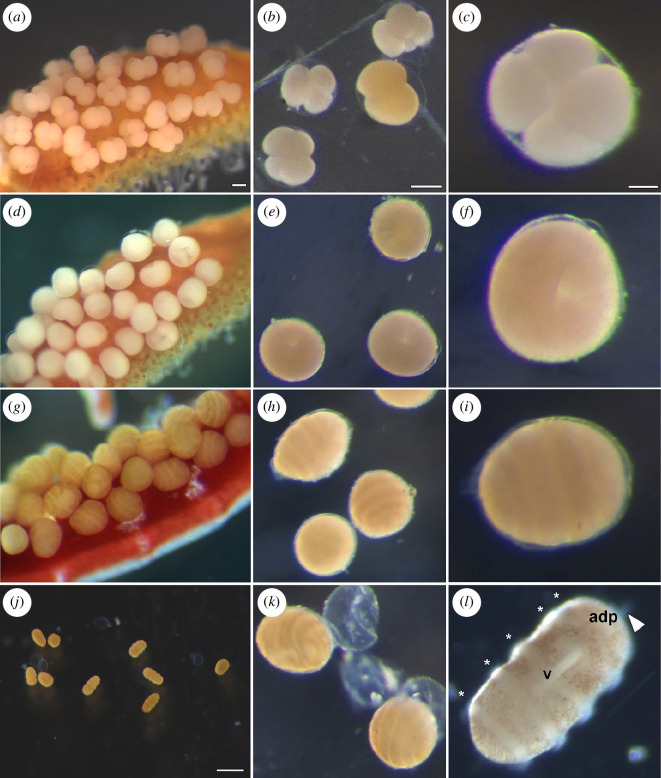
*Antedon mediterranea* embryogenesis on parental pinnules and *in vitro*. Embryos attached to adult genital pinnules (*a, d and g*) develop synchronously with those maintained *in vitro* (*b,c,e,f,h-l*). (*a–c*) 4-cell stage; (*d–f*) gastrula stage (24 hpf); (*g–i*) pre-hatching larva (72 hpf); (*j–l*) hatching (100 hpf) (*k*) and swimming doliolaria larva (*j,l*). * indicates ciliary band; arrowhead, apical tuft; adp, adhesive pit; v, vestibulum. Scale bar in (*a*) = 100 µm (same for *d,g*); scale bar in (*b*) = 100 µm (same for *e,h,k*); scale bar in (*c*) = 50 µm (applies to *f,i,l*); scale bar in (*j*) = 500 µm.

We found that the most efficient way to induce spawning of *A. mediterranea* adults in captivity required the exposure of ripe animals to external stressors, in particular to strong light (by means of a flashlight). During the reproductive season, we observed that male individuals were particularly sensitive to light and promptly responded to light cues by releasing sperm into the water. Female spawning generally occurred after sperm was released by males. Gamete spawning and fertilization took place within a few minutes following stimulation. While embryos normally remained attached to the genital pinnules of the female (figure 1), we found that it was possible to induce the release of hundreds of zygotes from the genital pinnules by transferring the female into a small glass dish (100 ml) filled with filtered seawater for about half an hour. Embryos were then collected from the bottom of the dish and transferred into plastic Petri dishes containing filtered seawater (see §5 for details), where they developed normally (figure 1). Frequent water changes and prompt removal of dead specimens were key to ensure survival and good health of the embryo cultures, which reached the swimming doliolaria stage (figure 1) after about 100 hpf (hours post-fertilization) (at 17 ± 1°C; figure 1*j–l*), with a hatching rate of 92.5 ± 1.8%. To ensure that the isolation of the embryos from the mother did not have a negative impact on their development, we jointly monitored the development of embryos that grew attached to the genital pinnules (figure 1*a,d,g*) and those raised in Petri dishes (figure 1*b,c,e,f,h,i*). We found no differences between embryos reared *in vitro* and those on the genital pinnules, with both reaching the swimming doliolaria stage at the same time.

### 2.2. Timing and main features of *A. mediterranea* developmental stages

**Figure 2.**
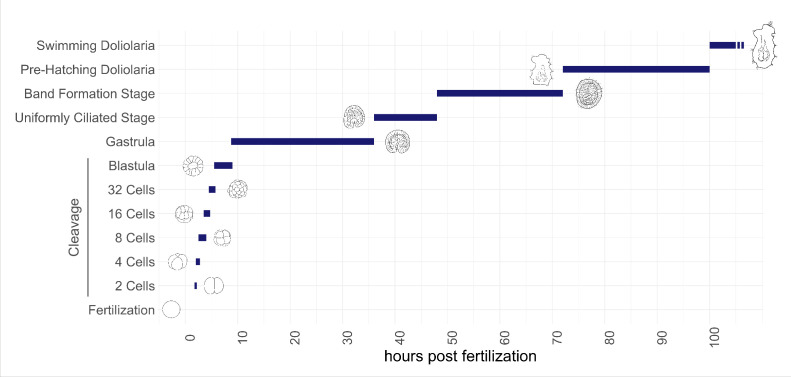
Developmental timeline of *Antedon mediterranea* embryogenesis. Graphical representation of *A. mediterranea* development at 17 ± 1°C: zygote stage (fertilization to 2 hpf), 2-cell stage (2–2.5 hpf), 4-cell stage (2.5–3.5 hpf), eight-cell stage (3.5–4 hpf), 16-cell stage (4–5 hpf), 32-cell stage (5–6 hpf), blastula stage (6–9 hpf), gastrula (9–36 hpf), uniformly ciliated stage (36–48 hpf), band formation stage (48–72 hpf), pre-hatching doliolaria stage (72–100 hpf) and swimming doliolaria stage (≥100 hpf).

The embryogenesis of *A. mediterranea* was previously described in classical microscopy studies of the late nineteenth and early twentieth centuries, often accompanied by beautiful drawings of selected developmental stages [15,16,33–35]. Here, we expand on these early reports by characterizing the main embryonic events, up to larval hatching, using modern microscopy techniques and by providing a standardized staging system of *A. mediterranea* embryogenesis based on easily identifiable developmental features. In parallel, we characterize the pattern of cell divisions in post-cleavage stages, using a newly optimized protocol of whole-mount immunofluorescence to label mitotic nuclei with an antibody against phosphorylated histone 3 (PhH3) (electronic supplementary material, figure S2) [36]. Overall, our standardized staging system includes 12 pre-metamorphic developmental stages including a zygote stage (fertilization to approximately 2 hpf), cleavage stages (2–6 hpf), a blastula stage (6–9 hpf), a gastrulation stage (9–36 hpf), a uniformly ciliated stage (36–48 hpf), a band formation stage (48–72 hpf), a pre-hatching doliolaria stage (72–100 hpf) and a swimming doliolaria stage (≥100 hpf) (figure 2).

**Figure 3.**
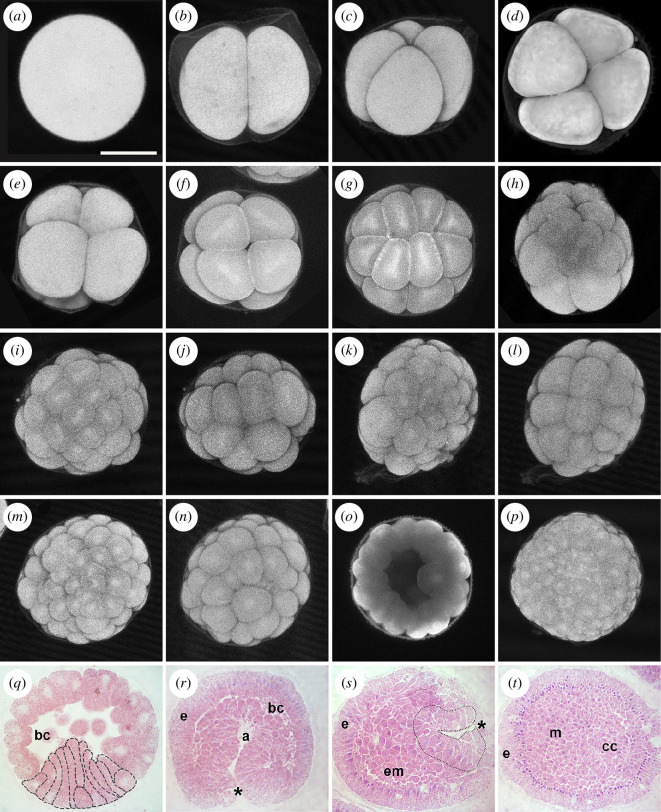
Morphological characterization of *Antedon mediterranea* embryogenesis. (*a–p*) Confocal z-projections of embryos labelled with phalloidin during the cleavage period: (*a*) zygote (1 hpf); (*b*) 2-cell stage (2 hpf); (*c,d*) 4-cell stage (2.5 hpf), in lateral (*c*) and frontal view (*d*); (*e,f*) 8-cell stage (3.5 hpf), in lateral view with animal pole on the top (*e*) and animal view (*f*); (*g*) 16-cell stage (4 hpf), in animal view; (*h*) 24-cell stage (4.5 hpf), in lateral view with animal pole on the top; (*i,j*) the two sides of a 32-cell stage (5 hpf); (*k, l*) the two sides of a 48-cell stage (5.5 hpf); (*m,n*) the two sides of a 64-cell stage (6 hpf); (*o*) mid-sagittal optical section of a blastula stage (6.5 hpf), showing the internal cavity; (*p*) initial gastrula (9 hpf). (*q–t*) Light microscopy of *A. mediterranea* embryos undergoing gastrulation: (*q*) sagittal section of an initial gastrula (9 hpf) with elongating cells at the vegetal pole (outlined with dashed lines); (*r*) sagittal section of a mid-gastrula (20 hpf), with asterisk indicating the blastopore at the vegetal pole; (*s*) late gastrula (36 hpf), the blastopore is closed and the archenteron is narrow (outlined with dotted line); (*t*) ciliated larva (48 hpf) with coelomic cavities. a, archenteron; bc, blastocoel; cc, coelomic cavities; e, ectoderm; em, ento-mesoderm; m, mesoderm. Scale bar in (*a*) = 100 µm (applies to all images).

*Zygote stage* (fertilization to approximately 2 hpf at 17 ± 1°C). The mesolecithal egg was spherical and about 220 µm in diameter (figure 3*a*). Upon fertilization, an ornamented membrane covered the embryo until hatching. This membrane appeared spiny by light microscopy but scanning electron microscopy (SEM) analysis revealed that the structure was folded in thin, irregularly distributed ridges along the surface (electronic supplementary material, figure S3).

*Cleavage and blastula stages* (approximately 2–9 hpf at 17 ± 1°C). The first cell division occurred at approximately 2 hpf and split the zygote into two nearly equal blastomeres (figure 3*b*). The second cleavage plane was meridian to the first and resulted in four cells of similar size (figure 3*c,d*; approximately 2.5 hpf). The third mitotic division separated animal and vegetal poles. The cleavage plane was parallel to the equator but closer to the animal pole, thus dividing the embryo into four smaller (animal) and four larger (vegetal) blastomeres (figure 3*e,f*; approximately 3.5 hpf). The next divisions occurred first at the animal and then at the vegetal pole. At about 4 hpf, most embryos reached the 16-cell stage, which consisted of eight smaller animal and eight larger vegetal cells (figure 3*g*). The following cleavage plane ran equatorially and divided the eight animal cells into 16 animal blastomeres of almost equal size, leading to a 24-cell stage, which was composed of eight large vegetal cells and two stacks of eight small animal cells (figure 3*h*). At about 5 hpf, the division of the vegetal blastomeres was completed and the embryo reached the 32-cell stage (figure 3*i,j*). Rearrangements of cell positions subsequently occurred, resulting in a wide blastocoel that started to form. The following division at the animal pole led to an almost spherical embryo of 48 cells (figure 3*k,l*), which soon thereafter reached the 64-cell stage (figure 3*m*,*n*; approximately 6 hpf). This corresponded to the beginning of the blastula period (figure 3*o,p* and electronic supplementary material, figure S3). During cleavage stages, cell divisions were generally characterized by a marked asynchrony between the animal and the vegetal pole, with cells dividing faster on the animal side. Of note, starting from the first cell division, a moderate level of developmental asynchrony was observed among embryos of the same batch, with cell divisions not taking place at the exact same time from one embryo to another. This asynchrony between embryos became even more evident later on, i.e. from blastula stages onwards (figure 2).

*Gastrulation period* (approximately 9–36 hpf at 17 ± 1°C). At the beginning of gastrulation, the blastomeres at the vegetal pole of the embryo changed their morphology: the most vegetal columnar cells underwent apical constriction, elongating toward the blastocoel cavity and acquiring a flask shape (figure 3*p,q*). At about 10 hpf, invagination of the ento-mesodermal layer began at the vegetal side and, at 20 hpf, the embryos reached the mid-gastrula stage (figure 3*r*). At this stage, the ento-mesoderm extended through the blastocoel, almost reaching the ectodermal layer at the animal pole. At the same time, small mesenchymal cells detached from the ento-mesodermal layer and started filling the cavity of the blastocoel. The ectodermal cells retained their columnar shape, while ento-mesodermal blastomeres near the blastopore showed the characteristic flask shape (figure 3*r*). During the next few hours, mesenchyme cells continued to fill the embryo cavity and, at approximately 36 hpf, the blastopore was closed in most embryos, with the archenteron taking the shape of a narrow sac (figure 3*s*). During gastrulation, cells in active proliferation were distributed throughout the embryo (electronic supplementary material, figure S2*a*).

**Figure 4.**
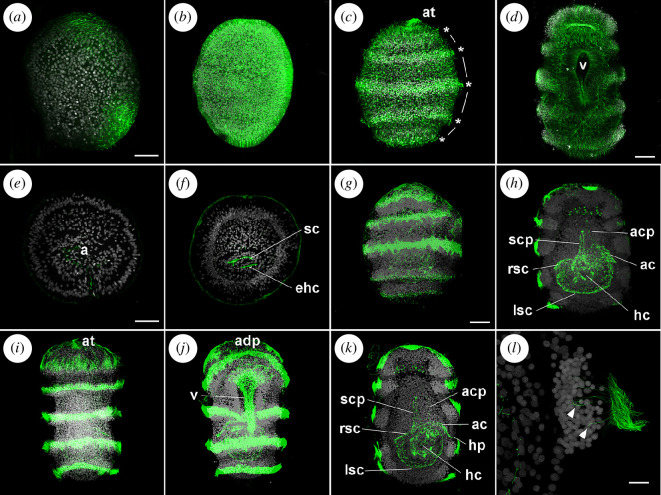
External and internal ciliogenesis in *Antedon mediterranea* embryos. Confocal z-projections of embryos immunolabelled with anti-β-tubulin antibodies (*a–d*) (green) or anti-acetylated α-tubulin antibodies (*e–l*) (green) and co-stained with 4′,6-diamidino-2-phenylindole (DAPI) (nuclei, grey). Maximum projections (*a–d,g,i,j*) and longitudinal sections (*e,f,h,k,l*) of representative samples. Anti-β-tubulin antibodies marked developing cilia in late gastrula (*a*), uniformly ciliated larva (*b*), band formation (*c*) and swimming doliolaria (*d*) stages. (*a–d*) are ventral views. Asterisks and lines in (*c*) indicate band and interband cells, respectively. Cilia in the ectoderm and in coelomic cavities were also immunoreactive to anti-acetylated α-tubulin in gastrula (*e*), uniformly ciliated larva (*f*), pre-hatching larva (*g*) and swimming doliolaria (*i–k*); (*e,g,i*) are dorsal views, (*h,k*) are longitudinal views and (*j*) is a ventral view. Magnification of a ciliated band in a swimming doliolaria larva is shown in (*l*); cilia departing from inner cells are indicated (arrowheads). ac, axocoel; acp, axocoel projection; adp, adhesive pit; at, apical tuft; ehc, enterohydrocoel; hc, hydrocoel; hp, hydropore; lsc, left somatocoel; rsc, right somatocoel; sc, somatocoel; scp, somatocoel projection; v, vestibulum. Scale bars in (*a,d,e,g*) = 50 µm (apply to *a–k*); scale bar in (*l*) = 10 µm.

*Uniformly ciliated stage* (approximately 36–48 hpf at 17 ± 1°C). The formation of mesenchyme continued and the blastocoel was no longer distinguishable (figure 3*t*). At the posterior end of the embryo, the archenteron split into two vesicles, the enterohydrocoel and the somatocoel, before the hydrocoel and the axocoel separated from the enteric sac (figures 3*t* and 4*f*). Together with the development of the coelomic cavities, ectodermal cells spread around the entire embryo and completed their transition into a uniformly ciliated epithelium (figure 4*b*). At this stage, immunoreactivity for the proliferation marker PhH3 was more concentrated in ectodermal cells (electronic supplementary material, figure S2*b*). The ectoderm of these embryos consisted of columnar cells regularly arranged around the mesodermal tissue (figure 3*t*). Most of these cells were further characterized by resting nuclei located basally, while mitotic signals were consistently detected in cells with nuclei localized in a central or apical region of the cell (electronic supplementary material, figure S2*b*).

*Band formation stage* (approximately 48–72 hpf at 17 ± 1°C). During this developmental stage, the characteristic five ciliary bands of the doliolaria larva started to form (figure 4*c*). At approximately 48 hpf, before the development of the band-interband pattern, some morphological changes in the epithelial cells were already detectable: future band cells became thin and tightly packed, while future interband cells remained larger (figure 4*c*). These interband cells gradually lost their cilia, and the epithelium of the embryo acquired the alternation of ciliated and non-ciliated domains typical of the doliolaria larva.

*Pre-hatching doliolaria stage* (approximately 72–100 hpf at 17 ± 1°C). Although still roundish in shape and enclosed in the fertilization membrane, the larva appeared almost completely formed. Under the fertilization membrane, the main larval features were easily discernable and included five transverse ciliary bands and two ventral grooves: the adhesive pit and the vestibulum (figures 1*g–i* and 4*d,j*). Cell proliferation was concentrated at the level of the developing transverse ciliary bands and the apical tuft and pit (electronic supplementary material, figures S1 and S2*c*), with PhH3-positive nuclei being localized on the external surface of the embryo. In internal tissues, mitotic nuclei were also observed in both mesenchymal tissues and coelomic cavities (electronic supplementary material, figure S2*d*).

*Swimming doliolaria stage* (≥100 hpf at 17 ± 1°C). After hatching (see figure 1*k*), the doliolaria larva began swimming in the water column. The swimming doliolaria appeared as a barrel-shaped larva that was 400–450 µm long. The larva was characterized by five ciliary bands that ran transversely along the body and by a long anterior tuft emerging from the apical pit. Ventrally, two grooves were observable, the anterior adhesive pit, used by the swimming doliolaria to attach to the substrate at the beginning of metamorphosis, and the vestibulum covered by cilia (figures 1*l* and 4*i,j*).

### 2.3. Ciliogenesis

To better characterize ciliogenesis during *A. mediterranea* embryogenesis, antibodies against β-tubulin (figure 4*a–d*) and acetylated α-tubulin (figure 4*e–l*) were used to label cilia at different stages. Cilia first appeared during gastrulation, at 26 hpf (figure 4*e*). At this stage, acetylated α-tubulin-positive cilia were observed in invaginating ento-mesodermal cells, projecting towards the developing archenteron (figure 4*e*). Later, at approximately 32 hpf, ciliogenesis began on ectodermal cells, so that, at 36 hpf, two undetermined areas of ciliated ectoderm, one anterior and one posterior were present in most embryos (figure 4*a*). At approximately 48 hpf, ciliogenesis extended throughout the ectoderm, with short and motile cilia covering the complete outer surface of the now uniformly ciliated embryo (figure 4*b*). Inside the embryo, a ciliated epithelium further bordered the developing coelomic cavities and the enterohydrocoel (figure 4*f*). During the band formation stage (figure 4*c*), the change of the uniformly ciliated ectoderm into an alternation of band and interband domains occurred. This was a progressive and complex process that led to the development of the characteristic ciliated bands of doliolaria larvae (figure 4*d*). A clear band-interband pattern was already observable in pre-hatching larvae, in which cilia were strongly marked by the acetylated α-tubulin antibody (figure 4*g,h*). In the swimming doliolaria larvae, intense staining was detected in the five ciliated bands, in the apical tuft, in the ventral ciliated grove and in the vestibulum (figure 4*i,j* and electronic supplementary material, video S1). Magnification of the band domain revealed further that not all ciliated cells faced the external surface of the larva, but some were internal with cilia projecting outside (figure 4*l* and electronic supplementary material, video S2). Inside the larva, the development of the coelomic cavities advanced, and the ciliated epithelium of the recently formed hydrocoel and axocoel as well as that of the right and the left somatocoels were strongly labelled (figure 4*h,k*). The axocoel branched into a thin projection directed anteriorly, while the right somatocoel extended along the dorsal midline of the larva (figure 4*h*).

### 2.4. Endoskeleton

**Figure 5.**
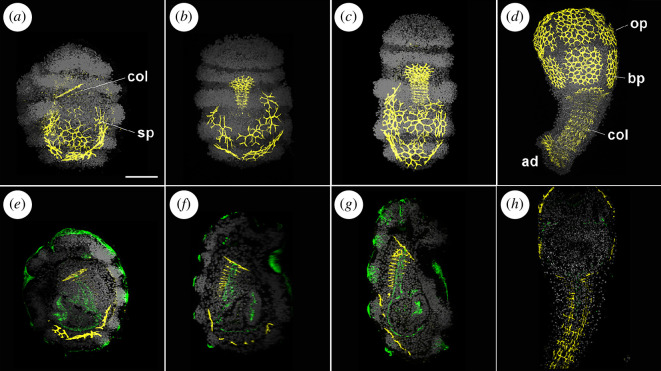
Ossicle development in *Antedon mediterranea*. Confocal z-projections of specimens immunolabelled with antibodies against phosphorylated Smad1/5/8 (pSmad1/5/8) (yellow) and acetylated α-tubulin (green) and co-stained with DAPI (nuclei, grey). Maximum projections (*a–d*) and mid-sagittal sections (*e–h*) of pre-hatching doliolaria larvae, early (*a,e*) and late (*b,f*) stage, swimming doliolaria larvae (*c,g*) and of specimens at the cystidean stage (*d,h*). At all stages considered, the pSmad1/5/8 antibodies labelled the developing ossicles of the crinoid skeleton. ad, attachment disk; bp, basal plate; col, columnar ossicles; op, oral plate; sp, skeletal plates. Scale bar in (*a*) = 100 µm (applies to all images).

The elements that form the post-metamorphic skeleton are already present at the larval stage [4,17]. We found that antibodies against phosphorylated Smad1/5/8 (pSmad1/5/8; Cell Signaling Technology, 9511S) specifically stain skeletal ossicles in *A. mediterranea* (figure 5). While all ossicles were strongly immunoreactive to those antibodies during development, no other cellular structure was labelled, indicating that the pSmad1/5/8 antibodies recognize a different epitope within the crinoid skeleton, and can therefore be used to follow ossicle development. Pre-hatching larvae (72 hpf; figure 5*a,b*; electronic supplementary material, video S3) had numerous spicules and developing ossicles: the oral and basal plates of the calyx were already recognizable, while the columnar elements of the stalk were still forming. In some cases, the contours of the attachment disk were also discernable (figure 5*b*). The complexity of the ossicles was highly variable between samples, but skeletal rudiments were always present at the pre-hatching doliolaria stage. In swimming doliolariae (figure 5*c*; electronic supplementary material, video S4), oral and basal ossicles began to acquire their typical plate shape and started to form numerous stromata. Columnar stalk ossicles were well developed and the rudiment of the attachment disk was always observable. After metamorphosis, at the cystidean stage, the skeleton was well formed, comprising oral and basal plates, columnar stalk ossicles and the attachment disk (figure 5*d,h*). Double staining with anti-acetylated α-tubulin antibody facilitated localization and characterization of the ossicles (figure 5*e,h*). Oral and basal plates were distributed at the periphery of the mesoderm, surrounding the coelomic and enteric cavities (figure 5*e,g*). The columnar ossicles were positioned along the dorsal midline of the larva, with the right somatocoel running at the centre of the developing skeletal structure (figure 5*e,g*).

### 2.5. Development and organization of the larval nervous system

**Figure 6.**
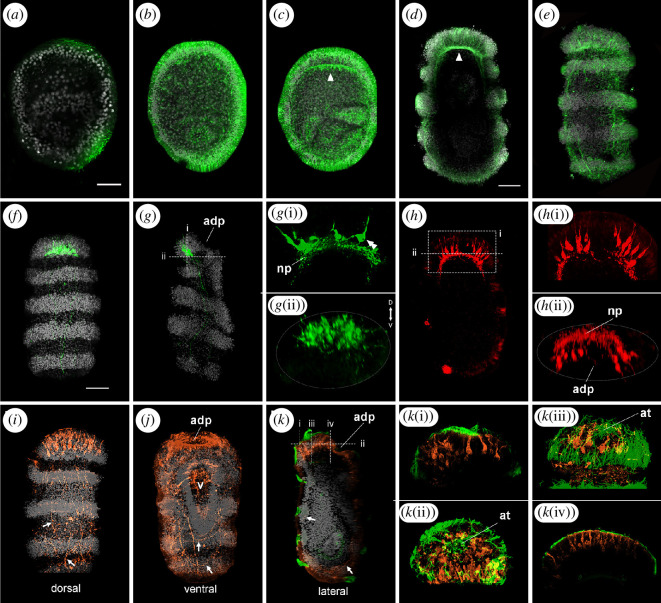
Development and cell type composition of the larval nervous system of *Antedon mediterranea*. Confocal z-projections of immunolabelled embryos and larvae showing axonal development (*a–e*) and neurotransmitter distribution (*f–k*) at several developmental stages. In most panels, nuclei are co-stained with DAPI (grey). While neuron projections are not detected at 36 hpf (late gastrula) (*a*) anti-β-tubulin antibodies (green) marked growing neurites at uniformly ciliated (*b*), band formation (*c*) and swimming doliolaria (*d,e*) stage. (*d*) is a longitudinal and (*e*) a dorsal view. In (*c,d*) the apical neural plexus is indicated with arrowheads. Serotonin (green) is localized in the anterior-dorsal apical organ and in dorsal and lateral neurites in swimming doliolaria larvae (*f, g*). (*f*) is a dorsal and (*g*) a lateral view. Apical organ cells are bottle-shaped, project into the anterior plexus (*g(i)*), and are concentrated dorsally (*g(ii)*). Double arrowheads in (*g(i)*) indicate a single bottle-shaped cell. GABA immunoreactivity (red) was detected anteriorly in swimming doliolaria larvae (*h*) (ventral view) in bottle-shaped cells that project locally into the apical plexus (*h(i)*) and were located ventrally around the adhesive pit (*h(ii)*). Double immunostaining for glutamate (orange) and acetylated α-tubulin (green) shows glutamatergic neurons and fibres anteriorly and along the body of the swimming doliolaria larvae. (*i*) is a dorsal, (*j*) a ventral and (*k*) a lateral view. Arrows indicate fibres and cells of the basiepithelial neural plexus. Antero-dorsally, large cells are arranged around the apical tuft (*k(i)–k(iii)*), while, ventrally, small cells are organized around the adhesive pit (*k(iv)*). adp, adhesive pit; at, apical tuft; D, dorsal; np, neural plexus; V, ventral; v, vestibulum. Scale bars in (*a,d,f*) = 50 µm (apply to all images).

While in *A. mediterranea* embryos the anti-acetylated α-tubulin antibodies were specific for cilia, anti-β-tubulin antibodies labelled both cilia and neural processes [4] and were thus used to specifically trace neural fibre outgrowth during development (figure 6*a,e*). Up to the uniformly ciliated stage (36–48 hpf), immunoreactivity was only observed in the cilia covering the surface of the embryo and bordering the internal coelomic/enteric cavities (figure 6*a,b*). Starting at 48 hpf, short nerve fibres running at the base of the epithelial cells were immunolabeled (figure 6*b*), indicating the presence of maturing neurons. As development proceeded, neural processes elongated to form a basiepithelial plexus that soon became thicker in the anterior portion of the embryo (figure 6*c*). In the swimming doliolaria, the neural plexus consisted of a complex network of nerve bundles running under the epidermis and towards the body surface. Anteriorly, the apical plexus consisted of densely packed fibres, with neural processes connecting to the apical pit (figure 6*d,e*).

We have previously performed immunohistochemistry on doliolaria sections showing that *A. mediterranea* larvae have a serotonergic apical organ [4], but its organization and the identity of neurons in the crinoid larval neural plexus remain unclear. With the newly optimized protocol for whole-mount immunohistochemistry, we examined the localization of three neurotransmitters (serotonin, GABA and glutamate) to reveal the architecture of the larval nervous system at the swimming doliolaria stage (figure 6*f–k*). Serotonin antibodies labelled a large cluster of approximatively 30 flask-shaped cells located on the anterior-dorsal side of the animal, at the level of the first ciliary band, just underneath the apical pit (figure 6*f,g*). The cell bodies were located deep within the ectoderm, above the thicker portion of the anterior neural plexus and were not directly associated with the ciliary epithelium. These serotonin-positive cells reached the anterior-dorsal surface of the embryo, where the apical pit is located, and their projections entered the anterior plexus and ran posteriorly along the dorsal and lateral sides of the larva (figure 6*g,g*(i,ii)). Interestingly, serotonin immunoreactivity started to be detected in a smaller group of cells already at the pre-hatching doliolaria stage (electronic supplementary material, figure S4). GABA-immunoreactive neurons were also found in the anterior portion of the larva, but were localized ventrally to the adhesive pit and organized into two clusters of about eight flask-shaped cells (figure 6*h,h*(i)). The apical portion of these neurons reached the surface ectoderm between the apical and adhesive pits, while axonal projections were local and terminated in the dense anterior neuropil (figure 6*h–h*(ii)). Anti-glutamate antibodies labelled a significant number of cells in the larva, more anteriorly than posteriorly (figure 6*i–k*). On the antero-dorsal surface, large flask-shaped cells surrounded the apical pit and tuft but did not appear to have extensive axonal projections (figure 6*i,k*(i–iii)). On the ventral side, numerous small bipolar cells were distributed along the edge of the adhesive pit (figure 6*j,k*(iv)). In addition, several glutamatergic neurons were stained in the neural plexus along the larval body, both dorsally and ventrally (figure 6*i,j*). These cells sent projections along the anterior–posterior axis, and, on the ventral side, axons appeared to loop around the posterior end of the larva. In summary, this analysis revealed the presence of a complex nervous system in the crinoid doliolaria, characterized already upon hatching by multiple distinct cell types.

### 2.6. Molecular patterning of the crinoid apical organ

**Figure 7.**
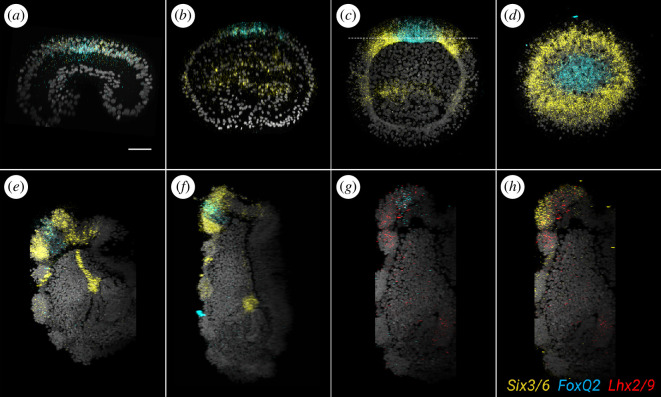
Molecular patterning of the apical region during *Antedon mediterranea* development. Confocal z-projections showing co-expression of *Ame_FoxQ2* (cyan), *Ame_Six3/6* (yellow) and *Ame_Lhx2/9* (red) during crinoid development using *in situ* HCR. All specimens were co-stained with DAPI (nuclei, grey). *Ame_Six3/6* and *Ame_FoxQ2* are co-expressed in the anterior/apical portion of the ectoderm at 20 hpf (early gastrula) (*a*), and 26 hpf (late gastrula) (*b*), while starting at the uniformly ciliated stage (*c,d*) (showing, respectively, a longitudinal and a dorsal view, the latter at the level of the dashed line in (*c*)) expression of the genes form two concentric domains that persist in the pre-hatching (*e*) and doliolaria (*f–h*) stages. At larval stage, *Ame_Lhx2/9* is co-expressed with both *Ame_FoxQ2* (*g*) and *Ame_Six3/6* (*h*). Scale bar in (*a*) = 50 µm (applies to all images).

The apical organ of eleutherozoan echinoderms is specified through a highly conserved gene regulatory network that is involved in patterning the anterior portion of the neuroectoderm [11,37–39]. In particular, early expression of transcription factors *Six3/6* and *FoxQ2* on the animal side of the embryo defines an area called the apical plate, in which downstream genes will determine the identity of apical organ neurons. To assess whether this network is also present in crinoids, we investigated the developmental expression of *Ame_Six3/6*, *Ame_FoxQ2* and *Ame_Lhx2/9* (one of the downstream genes known to be expressed in serotonergic neurons [40]). We first identified transcript sequences for *Ame_Six3/6*, *Ame_FoxQ2* and *Ame_Lhx2/9* in the *A. mediterranea* transcriptome [41] (electronic supplementary material, figure S5), and we then analysed their expression by optimizing a co-labelling protocol for *in situ* HCR [42] (figure 7). Five developmental stages were considered: two subsequent mid-gastrula stages (20 and 26 hpf at 17 ± 1°C), one uniformly ciliated stage (44 hpf at 17 ± 1°C), one pre-hatching doliolaria stage (72 hpf at 17 ± 1°C) and one swimming doliolaria stage (>100 hpf at 17 ± 1°C) (electronic supplementary material, figure S6). In embryos at 20 hpf, *Ame_FoxQ2* and *Ame_Six3/6* were co-expressed broadly on the anterior/animal side of the ectoderm (figure 7*a*). At the later mid-gastrula stage, the *Ame_FoxQ2* signal was already restricted to the animal pole, while *Ame_Six3/6* expression was still broad and overlapping with that of *Ame_FoxQ2* in the animal tip (figure 7*b*; electronic supplementary material, figure S6). Furthermore, *Ame_Six3/6* started to be expressed in ento-mesodermal cells at the tip of the archenteron cavity (figure 7*b*; electronic supplementary material, figure S6). In the uniformly ciliated stage, *Ame_FoxQ2* transcripts were still found at the animal tip of the embryo, but this region was now devoid of *Ame_Six3/6*, which instead formed a ring around the domain of *Ame_FoxQ2* expression (figure 7*c,d*). *Ame_Six3/6* was also expressed in the anterior wall of the enterohydrocoel (electronic supplementary material, figure S6).

In the pre-hatching doliolaria, the concentric expression of *Ame_FoxQ2* and *Ame_Six3/6* was still evident: *Ame_FoxQ2* labelled cells in the apical pit, while *Ame_Six3/6* expression was localized in the first ciliary band and in the developing adhesive pit (figure 7*e*). Weaker *Ame_Six3/6* expression was detected in the second and third ciliary bands, and a new cell population with strong *Ame_Six3/6* expression appeared just beneath the second ciliary band (figure 7*e* and electronic supplementary material, figure S6). At this stage, the downstream gene *Ame_Lhx2/9* was expressed on the dorsal side of the two ciliary bands and within the apical plate defined by *Six3/6* and *FoxQ2* (electronic supplementary material, figure S6). The expression of these genes remained similar in the swimming doliolaria larva. *Ame_FoxQ2* marked cells of the apical pit that also co-express *Ame_Lhx2/9* (figure 7*g*). *Ame_Six3/6* transcripts were distributed in the remaining anterior portion of the larva up to the third ciliary band, and a group of cells conspicuously expressing *Ame_Six3/6* was located between the second and third bands (figure 7*h* and electronic supplementary material, figure S6). On the dorsal surface up to the second ciliary band, *Ame_Six3/6* was co-expressed with *Ame_Lhx2/9* (figure 7*h*). Inside the larva, *Ame_Six3/6* labelled the axocoel, including the anterior projection of the coelom that reaches the anterior tip of the mesenchyme, while *Ame_Lhx2/9* was localized in the hydrocoel (electronic supplementary material, figure S6).

In other echinoderms, the network controlling anterior neuro-ectoderm formation is maintained on the animal side of the embryo through inhibitory interactions of Wnt signalling operating in the vegetal side [43]. Consistently, we found *Ame_Wnt8* expressed around the blastopore and in the posterior ectoderm throughout gastrulation and at the uniformly ciliated stage, while no expression was detected at later stages (electronic supplementary material, figure S6).

### 2.7. Development of the post-metamorphic nervous system

**Figure 8.**
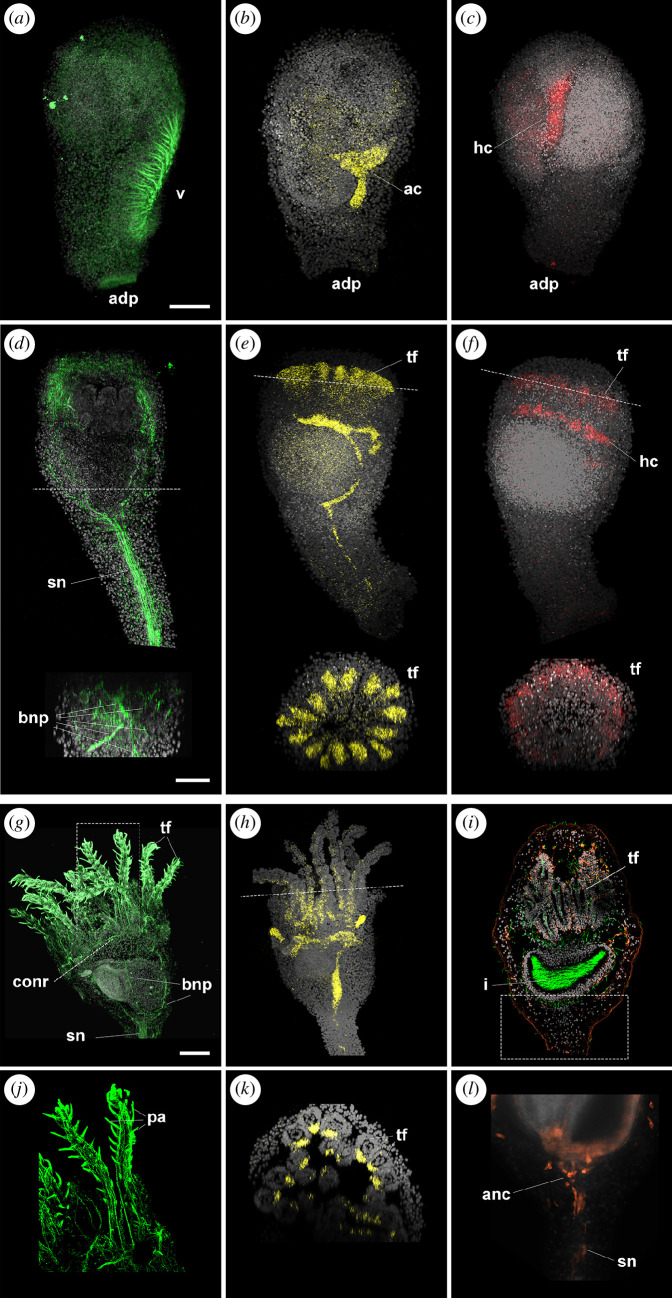
Formation of the post-metamorphic nervous system in *Antedon mediterranea*. Confocal z-projections of specimens immunolabelled with anti-β-tubulin (green) antibodies (*a,d,g,j*), or anti-glutamate (orange) and anti-acetylated α-tubulin (green) antibodies (*i,l*), or labelled with probes against *Ame_Six3/6* (yellow) (*b,e,h,k*) and *Ame_Lhx2/9* (red) (*c,f*). All specimens are co-stained with DAPI (nuclei, grey). Signal for all genes disappears in the apical region after settlement (*a–c*). At the cystidean stage (*d–f*), the post-metamorphic nervous system forms: neurites are labelled with β-tubulin (*d*) and the tube feet ectoderm starts to express *Ame_Six3/6* (*e*) and *Ame_Lhx2/9* (*f*). Transverse sections for each image are shown at the level indicated by the dashed lines. At the pentacrinoid stage (*g-l*), β-tubulin marks neural fibres in the stalk nerve, radial nerve primordia, circumoral nerve ring and tube feet, including the papillae (*g,j*); *Ame_Six3/6* is expressed on the oral side of the tube feet ectoderm (*h,k*); glutamate is localized in tube feet, neurons of the epidermal plexus, aboral nerve centre and stalk nerve (*i,l*), while acetylated α-tubulin shows cilia in the hydrocoel of the tube feet, the somatocoel and the intestine (*i*). Panels (*j*), (*k*) and (*l*) show specific portions of the pentacrinoid indicated by dashed lines/boxes in, respectively, (*g*), (*h*), and (*i*). ac, axocoel; adp, adhesive pit; anc, aboral nerve centre; bnp, brachial nerve primordia; conr, circumoral nerve ring; hc, hydrocoel; i, intestine; pa, papillae; sn, stalk nerve; tf, tube feet; v, vestibulum. Scale bars in (*a,d,g*) = 50 µm (applies to all images).

Recent studies have started to investigate the composition of the complex adult nervous system of eleutherozoan echinoderms [14,44], but its developmental origin still remains elusive. Crinoids have a gradual metamorphosis in which larval tissues are rearranged to form the adult body. This feature of the crinoid life cycle might help to understand the formation of the enigmatic adult body plan of echinoderms, including its nervous system. Previous studies have suggested that crinoid larval neurons degenerate at metamorphosis and that a tripartite nervous system develops in the adult [16,19]. The adult tripartite nervous system of crinoids consists of ecto-, hypo- and ento-neural components. Ecto-neural and hypo-neural nervous systems are also present in other echinoderms, extending, respectively, on the oral side along the midline and on each side of the ambulacral groove. In addition, crinoids possess a conspicuous aboral ento-neural nervous system, located within the ossicles of the calyx and arms (as well as in the stalk in sea lilies). During metamorphosis of *A. mediterranea*, we found that fibres of the apical neural plexus labelled by β-tubulin were quickly lost in settled larvae (see figure 8*a* at the level of the adhesive pit attached to the substrate). The ciliary bands also disappeared after settlement, while cilia could be occasionally observed in the vestibular ectoderm (figure 8*a*). Moreover, the expression of *Ame_FoxQ2* (data now shown), *Ame_Six3/6* and *Ame_Lhx2/9* disappeared from the apical surface, which was now attached to the substrate and became the aboral side of the animal (figure 8*b,c*). Conversely, *Ame_Six3/6* and *Ame_Lhx2/9* transcripts were still detected in the axocoel and hydrocoel, respectively (figure 8*b,c*; electronic supplementary material, figure S7*a*–*c*).

As metamorphosis progressed, the internal organs underwent a 90° rotation, after which the vestibulum of the swimming doliolaria larva reached the oral surface (corresponding to the posterior side of the doliolaria) at the cystidean stage (electronic supplementary material, figure S7*d*–*f*). The ciliated hydrocoel was positioned under the oral (vestibular) ectoderm, and, from it, five sets of three tube feet primordia emerged (as shown by acetylated α-tubulin immunoreactivity) (electronic supplementary material, figure S7*d*). Moreover, the post-metamorphic nervous system started to develop. Antibodies directed against β-tubulin labelled a prominent stalk nerve already in cystidean samples, indicating that the ento-neural system differentiated shortly after metamorphosis (figure 8*d*). At late cystidean stages, fibres also directed orally from the aboral nerve centre (figure 8*d*). Interestingly, already at cystidean stages, the oral ectoderm started to express both *Ame_Six3/6* and *Ame_Lhx2/9* at the level of the tube feet primordia (figure 8*e,f*; electronic supplementary material, figure S7*e*,*f*).

It has previously been shown that the pentacrinoid nervous system comprises a complex basiepithelial nerve net of serotonergic, GABAergic and peptidergic neurons, concentrated at the level of the tube feet, and a conspicuous ento-neural system comprising cholinergic and peptidergic cells [4,26]. Immunoreactivity for β-tubulin showed that the post-metamorphic nervous system was highly interconnected, with five bundles of fibres directing from the ento-neural aboral nerve centre to a circumoral nerve ring, which was in turn connected to fibres that run in the tube feet (figure 8*g,j*). Curiously, the tube feet expression of *Ame_Six3/6* persisted in later pentacrinoid stages, but was restricted to the internal portion of each tube feet, facing the mouth (figure 8*h,k*).

We further expanded this analysis by characterizing the localization of glutamatergic neurons (figure 8*i,l*). A significant number of glutamate-positive cells were detected in the tube feet (figure 8*i*; electronic supplementary material, figure S7*g*). Contrary to the serotonergic neurons, these glutamatergic cells did not form an obvious axonal net, with immunoreactivity found mainly within cell bodies. Interestingly, glutamate labelling was also found at the tip of the papillae (electronic supplementary material, figure S7*g*). Scattered glutamatergic neurons were also identified across the calyx epidermis (figure 8*i*). These cells appeared to be more concentrated on the aboral side and to possess short axonal projections. A variable number of neurons were also labelled in the ento-neural system (figure 8*l*). A few glutamate-positive cells were further visible just oral to the aboral nerve centre, while several glutamatergic fibres and cell bodies were detectable along the stalk nerve (figure 8*l*; electronic supplementary material, figure S7*g*).

## 3. Discussion

Although crinoids occupy a key phylogenetic position as basally branching echinoderms [6], their development has been investigated less than that of other echinoderm groups, and the molecular mechanisms regulating their embryogenesis have started to be considered only very recently [4,5,21]. The difficulty in collection and maintenance under laboratory conditions of many crinoid species, the scarce information about cues governing spawning and reproduction, and the limited availability of techniques to investigate the molecular control of development have hampered the use of crinoid models in developmental and evolutionary studies [16,17,27–29]. In this work, we show how to overcome most of these obstacles, as *A. mediterranea* can be found in shallow waters during its reproductive season, adults can be easily collected and gamete release can be induced in captivity to obtain hundreds of fertilized eggs. Although *A. mediterranea* is an externally brooding species [32], and embryos normally develop attached to the genital pinnules of the female [4,17], our methods allowed us to detach embryos from genital pinnules and successfully rear them *in vitro* to precisely follow embryogenesis. Furthermore, we have optimized techniques for whole-mount immunohistochemistry and multiplexed *in situ* HCR that allowed us to monitor development and characterize embryonic, larval and post-metamorphic structures to better understand the morphology of this enigmatic group of echinoderms.

### 3.1. Crinoid embryogenesis and formation of larval structures

Our current understanding of the early development of crinoids is based on old microscopy work from the late nineteenth and early twentieth century [15,16]. These studies reported detailed descriptions of the cellular and subcellular modifications taking place during embryonic development [33–35], but standardized developmental timing and staging under controlled laboratory conditions were lacking. Here, we followed *A. mediterranea* embryogenesis *in vitro* and described each embryonic phase together with its developmental timing using high-resolution images. Interestingly, we found no differences in survival, embryo morphology and developmental timing between embryos developing *in vitro* or on genital pinnules, suggesting that maternal contribution after spawning might be limited to preventing predation and maintaining access to clean and oxygenated seawater. Consistent with previous descriptions of crinoid development, we found that cleavage in *A. mediterranea* was holoblastic, radial and unequal, forming smaller animal blastomeres and larger vegetal blastomeres from the 8-cell stage. Cell divisions were asynchronous and occurred faster in animal blastomeres, resulting in an irregularly shaped embryo and cell divisions that were difficult to follow. Curiously, we also observed that embryos of the same batch developed asynchronously both *in vitro* and on genital pinnules. These features were not related to our experimental conditions, as they have been reported previously [15,45] and hence appear to be a specific characteristic of feather star development.

Earlier microscopy-based studies reported that gastrulation in crinoids begins as unipolar ingression of single cells before gradually turning into an invagination process [15,16]. This was refuted in *Florometra serratissima* and in *A. japonica*, for which gastrulation by invagination [46,47] or by holoblastic involution [45] were proposed instead. According to our data, *A. mediterranea* development proceeds with gastrulation by invagination, preceded by shape changes of vegetal pole cells. These vegetal blastomeres gradually acquire a flask-like morphology, likely narrowing at the apical side. Apical constriction is a well-known mechanism that drives primary invagination in both sea urchins and amphibians [48–50] and might therefore be functional during bending of the ento-mesodermal layer in *A. mediterranea*. During gastrulation, the thick ento-mesodermal layer quickly reaches the animal pole, which is accompanied by a drastic reduction of blastocoel size, which in turn is progressively filled by mesenchymal cells, as previously shown in other crinoids [45,46]. This contrasts with the initial phases of gastrulation in sea urchins where the ento-mesoderm only occupies a small portion of the embryonic cavity [48]. The increase in embryo complexity during and after gastrulation is accompanied by widespread proliferation. A peculiar pattern of cell division was observed in the ectoderm during gastrulation, with nuclei of dividing cells being concentrated on the apical surface. This condition is similar to interkinetic nuclear migration, which is characterized by the migration of nuclei along the apical-basal length of the cell during the cell cycle and which has been observed in the vertebrate neural plate as well as in other pseudostratified epithelia [51].

One of the hallmarks of echinoderm larvae is the presence of ectodermal ciliary bands. Previous studies have highlighted that, in many crinoid species, the ciliary bands of the doliolaria are derived from a uniformly ciliated ectoderm [17,23,47]. In *A. mediterranea,* both ento-mesodermal and ectodermal tissues are highly ciliated. The formation of ectodermal cilia started at around 32 hpf in two domains, one located near the animal pole and one more posteriorly, and then expanded through the ectoderm, so that, by 48 hpf, a uniformly ciliated larva started to rotate within the fertilization membrane. This mechanism of cilia formation has previously been reported, at the same developmental stage and with similar dynamics, in another feather star, *F. serratissima*, where the first ectodermal motile cilia form at the animal pole and then progressively expand along the surface of the embryo [47]. As described in *F. serratissima*, the band-interband pattern of the epidermis of the *A. mediterranea* doliolaria formed by a combination of proliferative events and morphological changes in cell shape and arrangement. In the developing ciliary bands, cells became smaller and more tightly packed, probably owing to the extensive proliferation in this region of the pre-hatching doliolaria. In comparison, only few dividing cells were detected in the interband domains where cells remained large and lost their cilia. The pre-hatching doliolaria already showed well-developed transverse ciliary bands, an apical tuft and two ventral ciliated grooves: the vestibulum and the adhesive pit.

We found that the use of antibodies against acetylated α-tubulin and pSmad1/5/8 was instrumental for describing the formation of internal structures during larval development, including the coelomic and enteric cavities and the skeleton. After invagination, a ciliated epithelium bordered the enteric sac, which separated into an anterior entero-hydrocoel and a posterior somatocoel at the uniformly ciliated stage. The entero-hydrocoel subsequently elongated, acquiring a dumbbell-like shape, while the somatocoel curved around it, assuming a crescent shape. By the pre-hatching doliolaria stage, the coelomic cavities and the enteric sac were formed. From this stage, acetylated α-tubulin staining also highlighted the presence of two anterior coelomic projections: one was part of the right somatocoel and was located within the rudiments of the stalk columnar ossicles, while the other (that was difficult to visualize by microscopy staining [4]) branched from the axocoel and almost reached the anterior ectoderm. Interestingly, several spicules and developing ossicles were already recognizable at the pre-hatching doliolaria stage. Rudiments of the future oral and basal plates of the post-metamorphic calyx were observed in the posterior portion of the larvae, surrounding the coelomic and enteric cavities, while columnar ossicles developed along the dorsal midline within the mesenchyme, surrounding the somatocoel projection. The oral and basal plates were already arranged in a pentaradial pattern and remained in a similar position after metamorphosis. This represents the first structure of the crinoid body plan to acquire pentaradial symmetry, followed by the hydrocoel in the settled doliolaria stage, suggesting that the molecular programs that specify pentaradial symmetric patterns in the ento-mesoderm are active before metamorphosis. Primordia of the skeletal plates have already been identified in doliolaria larvae of different feather star species [47,52,53]. A high variability in ossicle number and complexity was previously reported in *F. serratissima* larvae, in which ossicles first appear between initial and late doliolaria stages [52]. Conversely, in *A. mediterranea*, numerous ossicles were already present in pre-hatching larvae, suggesting that skeletogenesis was initiated during early development. Taken together, these results complement previous descriptions of coelomic and skeletal development in other crinoid species [15,16], contribute to highlight the differences between feather stars and sea lilies [15,16,21,33–35,52], and indicate that immunohistochemistry is a valuable tool for understanding the development of crinoid tissues.

### 3.2. Cell type diversity and organization of the crinoid larval nervous system

Previous studies highlighted the presence of serotonergic neurons in the anterior ectoderm of crinoid larvae [4,17,19]. However, technical limitations of existing protocols, mostly restricted to tissue sections and without the possibility to test gene co-expression, together with scant data on embryogenesis, have impeded the description of the early development and three-dimensional organization of the larval nervous system. Here, using newly optimized protocols for whole-mount immunohistochemistry and *in situ* HCR, we provide a more comprehensive characterization of the crinoid larval nervous system and its molecular specification. Short neural fibres were first detected at the base of the epidermis at 48 hpf and later formed an intricate basiepithelial plexus. In the pre-hatching doliolaria, the nervous system consisted of a network of nerve bundles extending under the epidermis with a thickening in the anterior region, where serotonergic neurons were already visible. The latter finding is consistent with a previous report on the localization of serotonin in uniformly ciliated embryos of the sea lily [19].

**Figure 9.**
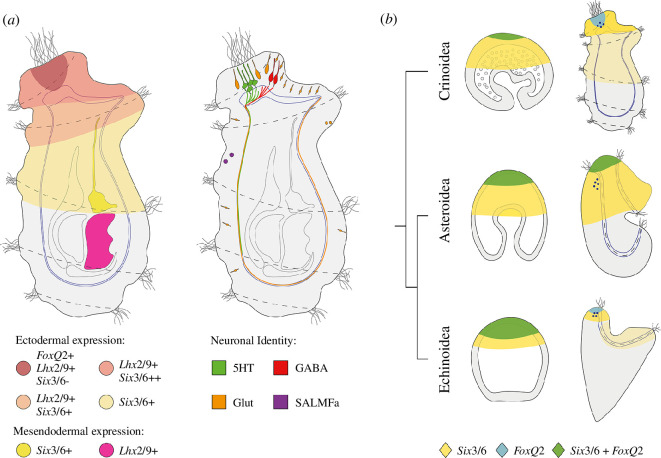
Comparison of echinoderm larval nervous system development. (*a*) Expression data and neural populations in the swimming doliolaria larva (lateral view). (*b*) Comparison of expression of *FoxQ2* and *Six3/6* across echinoderms. 5HT: serotonin; GABA: γ-aminobutyric acid; Glut: glutamate; SALMFa: SALMFamide neuropeptide.

In the swimming doliolaria, the larval nervous system was fully differentiated and consisted of a complex network of serotonergic, GABAergic and glutamatergic cells (figure 9*a*). A prominent apical organ was present in the anterior-dorsal region and was composed of about 30 serotonergic neurons. While GABAergic neurons appeared to form local connections only, both glutamatergic and serotonergic neurons were observed projecting posteriorly through the anterior neural plexus. Although circuit connectivity has not been addressed in this study, these findings suggest that serotonergic neurons, along with glutamatergic neurons, may play a role in facilitating long-distance communication between the apical organ and other areas of the sub-epithelial neural plexus.

The number of serotonergic cells varies greatly across echinoderms: while the starfish bipinnaria and the holothuroid auricularia larvae possess 30–50 and approximately 20 serotonergic neurons, respectively [11,54–57], early pluteus larvae of sea urchins have 4–6 serotonergic cells, with their number increasing as the larva grows [58]. This variability indicates that, while serotonergic neurons are a highly conserved feature of larval development in echinoderms, their number and distribution evolved independently in each echinoderm lineage. In addition to serotonergic cells, multiple large glutamatergic cells were present around the *A. mediterranea* apical pit, which might represent support cells often associated with apical organs [59]. The adhesive pit was further bordered by numerous small glutamatergic cells as well as by large GABAergic cells that sent projections to the anterior neural plexus. If serotonergic neurons of the apical organ have a sensory function, as described in other ciliated larvae [59], it is possible that they interact with these GABAergic neurons to direct settlement and metamorphosis. In fact, GABA has previously been shown to be involved in metamorphosis of sea urchin larvae [60].

While the apical organ had already been identified in crinoid larvae, neurons in the basiepithelial plexus remained uncharacterized. In addition to serotonergic projections from the apical organ, we found that the diffuse nerve plexus contained several glutamatergic neurons and fibres, organized both ventrally and dorsally. To our knowledge, this represents the first insight into the identity of basiepithelial neurons in crinoids and suggests that the nervous system of the swimming doliolaria larva has a large glutamatergic (and thus likely excitatory) component. Contrary to most other echinoderm larvae studied to date, the crinoid plexus does not appear to be associated with the ciliary bands. Instead, the nerve plexus is broadly distributed and axons travel throughout the entire ectoderm.

### 3.3. Conservation of apical organ development

The molecular control of apical organ development has been thoroughly investigated in several echinoderms, including sea urchins and starfish [40,61,62]. Different transcription factors, acting during gastrulation and organogenesis, have been shown to be required for the correct specification and positioning of the apical organ. Of these, *Six3/6* and *FoxQ2* are upstream regulatory components, as experimental knock-down of either one of these genes leads to the complete loss of anterior serotonergic neurons, while *Fezf*, *Nk2.1* and *Lhx2/9* are downstream effectors [40,63–65]. Comparative studies on the expression of these genes in multiple phyla have recently led to the hypothesis that a conserved anterior gene regulatory network (aGRN) might be involved in the specification of the anterior neuroectoderm across Bilateria [37,38,61,66]. However, there are significant differences in the organization of serotonergic neurons and the expression of aGRN transcription factors between echinoderm classes. For example, in starfish, serotonergic neurons are born throughout the apical surface of the larva and then migrate to form a dorsal bilateral structure within the ciliary band, while, in sea urchins, serotonergic cells develop only locally, in a region located dorsally to the ciliary band [11,54,58]. Moreover, in starfish, *FoxQ2* and *Six3/6* are co-expressed in the apical plate throughout development, while, in sea urchins, after early co-expression, the two genes form mutually exclusive concentric rings of expression (figure 9*b*) [40,63]. Previous chromogenic *in situ* hybridization analyses in sea lily and feather star larvae showed that *Six3/6* is expressed in the anterior portion of the ectoderm [5,20]. To explore the conservation of apical organ development in crinoids, we carried out the first multiplexed fluorescent *in situ* hybridization in crinoids, showing that co-expression of *Ame_FoxQ2* and *Ame_Six3/6* is already visible during gastrulation, in a broad anterior ectodermal domain (apical plate), and is subsequently restricted anteriorly. After gastrulation, *Ame_Six3/6* disappeared from the *Ame_FoxQ2* domain and formed a ring around the anterior tip of the animal, as in sea urchins. After the formation of the apical plate, the downstream gene *Ame_Lhx2/9*, which is involved in the formation of serotonergic neurons in starfish [40], started to be expressed within the first two ciliary bands and was co-expressed with *Ame_FoxQ2* anteriorly, in the area where serotonergic neurons form. Taken together, these results support the conservation of the aGRN in crinoids and indicate that the ancestral organization of the network in echinoderms included the formation of concentric rings of *Six3/6* and *FoxQ2* expression in the apical plate.

### 3.4. The crinoid post-metamorphic nervous system

The adult nervous system of echinoderms follows the pentaradial symmetry of the body, but its origin and early development have long remained obscure [67]. In crinoids, most of the larval tissues are maintained at post-metamorphic stages, but the doliolaria nervous system degenerates at metamorphosis, as we demonstrated with the loss of apical neurites and of the apical expression of aGRN genes shortly after settlement. We have previously shown that, at the post-metamorphic pentacrinoid stage, the nervous system already comprises ecto-neural and ento-neural components [4]. Here, we further revealed that these neurons begin to form at the cystidean stage, with neurites already being visible at the level of the developing tube feet, in the ento-neural stalk nerve, in the aboral nerve centre, and in the brachial nerve primordia. Moreover, we also discovered that the early tube feet ectoderm, where a concentrated net of serotonergic, GABAergic and glutamatergic neurons develops in the pentacrinoid [4], starts to express the anterior genes *Ame_Six3/6* and *Ame_Lhx2/9* already at the cystidean stage. A recent study has reported that the tube feet ectoderm of *A. japonica* cystideans additionally expresses *Otx* and *Pax4/*6 [5]. Taken together, these results indicate that the newly formed post-metamorphic ecto-neural system in crinoids expresses conserved anterior genes, consistent with what was recently discovered in eleutherozoans [44]. The inactivation of these genes during settlement and their re-activation in cystideans, however, suggests that this network is being redeployed secondarily at the adult stage, a process that might have evolved independently in different echinoderm groups.

## 4. Conclusions

The adult echinoderm body plan has long puzzled zoologists [16]. In order to understand the evolution of this phylum, and more broadly of deuterostomes, it is important to compare data from as many different echinoderm taxa as possible. Although crinoids are in an ideal phylogenetic position to investigate the evolution of this enigmatic phylum, biological and technical problems have hindered their study, resulting in far less information available for this group compared with other echinoderm groups. In this work, we developed methods for culturing *A. mediterranea* embryos *in vitro* and provided a standardized staging table for its embryogenesis, making early crinoid embryos and larvae accessible to experimental research. Our analysis also represents the most comprehensive description of the crinoid larval nervous system to date, revealing a complex organization and the presence of a significant number of morphologically and functionally diverse cell types. These results support the conservation of an aGRN controlling the formation of the apical organ in ambulacrarians. Overall, our work will contribute to the promotion of crinoids as model systems in developmental and evolutionary biology, allowing comparisons of crinoid development with that of other well-established animal models to help clarify the many unresolved questions about deuterostome evolution.

## 5. Methods

### 5.1. Animal collection and maintenance

*Antedon mediterranea* adults were collected during winter 2021 in the Gulf of La Spezia (Ligurian Sea, Italy). Animals were captured using fishing nets in shallow waters of between 20 cm and 2 m depth, with surface water temperatures ranging between 14°C and 18°C. Following capture, *A. mediterranea* adults were immediately transferred to the laboratory, where they were maintained in seawater aquaria with a closed multi-circulation system including mechanical, chemical and biological filters. The physical and chemical parameters of the seawater were verified regularly and adjusted if necessary [68]. The temperature was set to 17 ± 1°C, and natural light–dark conditions were implemented. Animals were fed with commercial food for filter-feeding marine animals (Coralific Delite) and the general fitness of the adults was monitored daily.

### 5.2. Embryo cultures

In captivity, mature animals of both sexes were kept in the same tank and were found to respond to external stressors by spawning. The most efficient external stressors to induce spawning were water turbulence and intense light (by use of a flashlight). Generally, male individuals spawned first, possibly stimulating mature females to release the eggs that were fertilized and retained on genital pinnules. To detach fertilized eggs from genital pinnules, gravid females were stressed, 15 min after spawning, by transferring them into 100 ml glass dishes containing seawater for 30 min, with occasional pipetting of seawater on the genital pinnules. Released zygotes were collected and transferred from the bottom of the glass dish to sterile plastic Petri dishes (100 embryos per dish). Dirt was carefully removed from the culture dishes, and embryos were reared in filtered seawater at 17 ± 1°C until they hatched. This procedure allowed the collection of hundreds of embryos, with many more remaining on the female genital pinnules. Females were subsequently moved back to the aquaria, and embryonic development on pinnules was monitored daily under a stereomicroscope until hatching. Embryos maintained *in vitro* were regularly checked as well, dead specimens were promptly removed and optimal culture conditions were maintained by a daily complete replacement of the seawater. The *in vitro* hatching rate was calculated for three batches of 100 embryos each as (number of swimming doliolariae larvae ÷ initial zygotes) × 100. During the whole culture period, embryos were fixed at different developmental stages (see §§5.3–5.5 for fixation details). During the first 12 hpf, embryos were fixed every 30 min. Subsequently, specimens were collected every hour between 20 and 36 hpf, and at 44, 48, 60, 72, 96 and 100 hpf.

### 5.3. Whole-mount phalloidin staining

To study cleavage, embryos from 1 to 12 hpf were processed for phalloidin staining. After fixation in 4% paraformaldehyde, 0.5 M NaCl and 0.1 M 3-(N-morpholino) propanesulfonic acid (MOPS) fixative (pH 7.5) for 90 min at room temperature, samples were washed in phosphate-buffered saline + 0.01% Triton X (PBST) and incubated in 1.5% H_2_O_2_, 5% formamide and 0.2× saline-sodium citrate (SSC) solution for 15 min. Permeabilization was performed in 1% DMSO in PBST for 15 min. Embryos were then washed in PBST and incubated for 2 h in 50% PBST/50% inactivated normal goat serum (NGS). Samples were subsequently stained at 4°C for two days with phalloidin-atto−488 (Sigma-Aldrich), diluted 1:50 in PBST, washed several times in PBST, mounted in 80% glycerol on microscope slides and examined with a Leica SP8 or a Nikon A1 laser scanning confocal microscope.

### 5.4. Whole-mount immunolocalization

In previous work, we designed a protocol for assaying immunofluorescence on paraffin sections of swimming doliolaria larvae [4]. Here, we optimized a new protocol for whole-mount immunofluorescence at different stages of *A. mediterranea* development. Embryos and larvae from the mid-gastrula to the swimming doliolaria stage were fixed in MOPS fixative and stored in 100% methanol at −20°C. Samples were washed with PBST and bleached in 1.5% H_2_O_2_, 5% formamide and 0.2× SSC. Bleaching was performed at room temperature under light for 35 min for embryos (up to the pre-hatching stage) and for 45 min for larvae (pre-hatching stage and swimming doliolaria larvae). Embryos were permeabilized by washing them with a permeabilization solution (1% DMSO in PBS + 1% TritonX) for 10 min and then left overnight in PBST at 4°C. Larvae were left overnight in permeabilization solution at 4°C, then further permeabilized with 2 µg ml^−1^ proteinase K for 10 min at 37°C and post-fixed in MOPS fixative for 30 min. Upon treatment, all samples were preincubated in 50% NGS in PBST for 2 h and incubated in the same solution with primary antibody (table 1). After several washes in PBST, embryos and larvae were preincubated in 1% BSA in PBST and incubated overnight at 4°C in PBST with the corresponding secondary antibody (table 1) and 4′,6-diamidino-2-phenylindole (DAPI) (1:1000) to label the nuclei. After PBST washes, the samples were mounted with : 1,4-diazabicyclo[2.2.2]octane (DABCO) or 80% glycerol on microscope slides and observed using a Nikon A1 laser scanning confocal microscope or an Olympus V3000 laser scanning confocal microscope.

**Table 1.** List of antibodies and their dilutions used in this study.

antigen	raised in	supplier	RRID	dilution
phosphorylated histone H3	rabbit	Abcam, ab5176	AB_304763	1:600
β-tubulin (clone 2−28−33)	mouse	Sigma-Aldrich, T5293	AB_477580	1:400
acetylated α-tubulin	mouse	Sigma-Aldrich, T6793	AB_477585	1:250
phosphorylated Smad1/5/8	rabbit	Cell Signaling, 9511S	AB_331671	1:100
serotonin	rabbit	Sigma, S5545	AB_477522	1:200
GABA	rabbit	Sigma, A2052	AB_477652	1:250
glutamate	rabbit	Sigma, G6642	AB_259946	1:500
rabbit IgG-488	chicken	Thermo Fisher	AB_143165	1:500
mouse IgG-568	goat	Thermo Fisher	AB_141371	1:500

### 5.5. Whole-mount *in situ* hybridization

Transcript sequences of *Ame_Six3/6*, *Ame_FoxQ2* and *Ame_Lhx2/9* were retrieved from the *A. mediterranea* transcriptome [41] using reciprocal best BLAST hits (electronic supplementary material, table S1). Gene orthology was assessed by phylogenetic analysis using SeaView [69]. Protein sequences were aligned using MUltiple Sequence Comparison by Log-Expectation (MUSCLE) [70], and phylogenetic trees were constructed using the Neighbour Joining method with 1000 bootstrap repetitions. Chromogenic *in situ* hybridization was performed as previously described [4]. Digoxigenin-labelled riboprobes specific for *Ame_FoxQ2* (Fwd: GCAATGGCAATTATGAACTCACCA; Rev: CCGTTAGCGCTTCGGCTTAT) were synthetized as in [71]. For *in situ* HCR, probes for *Ame_Six3/6*, *Ame_FoxQ2* and *Ame_Lhx2/9* were ordered from Molecular Instruments. A whole-mount HCR protocol was optimized for *A. mediterranea* embryonic, larval and post-metamorphic stages by combining aspects of an amphioxus HCR protocol [72] with the chromogenic *in situ* hybridization protocol previously published for this crinoid species [4]. All samples were rehydrated through a methanol/H_2_O series in Eppendorf tubes and then washed in PBST. Pentacrinoids were decalcified overnight in a solution of 5% EDTA in nuclease-free water (nfH_2_O). All samples were bleached in bleaching solution on aluminium foil. Swimming doliolaria larvae were bleached for 45 min, while embryonic and pentacrinoid stages were bleached for 20 min. While doliolaria larvae were incubated in permeabilization solution overnight at 4°C, embryonic and pentacrinoid stages were incubated for 15 and 30 min, respectively, and subsequently left in PBST overnight at 4°C. The following day, swimming doliolaria larvae were further permeabilized by proteinase K treatment at a concentration of 4 μg ml^−1^ at 37°C for 8 min followed by post-fixation in 4% PFA. All stages were pre-hybridized in hybridization buffer for 2 h at 37°C and then incubated with probes in hybridization buffer for 5 days at 37°C. After 6 days, the samples were washed in washing buffer followed by several washes in SSC + 0.1% TritonX (SSCT). Samples were then left in amplification buffer for 30 min and incubated with the hairpins for at least 20 h at room temperature and in the dark. All samples were subsequently washed in SSCT and incubated overnight with PBST + 1 μg ml^−1^ DAPI at 4°C in the dark. Samples were mounted in 100% glycerol in glass-bottom dishes and imaged using an Olympus V3000 inverted confocal scanning microscope.

### 5.6. Light microscopy

Embryos from 8 to 48 hpf were processed for standard light microscopy as previously described [73]. Briefly, samples were dehydrated through an ethanol series and embedded in Technovit resin (Heraeus Kulzer) according to manufacturer’s guidelines. Sections of 5 μm were cut with a microtome and stained with haematoxylin and eosin. Samples were observed under a Leica microscope and photographed using a Leica DFC‐320‐C camera.

### 5.7. Scanning electron microscopy

SEM analyses were performed as described in [74]. Briefly, zygotes were fixed in 2% glutaraldehyde in seawater for 2 h and washed overnight in filtered seawater at 4°C. Samples were post-fixed with 1% OsO_4_ in seawater and glucose for 2 h, washed in distilled water and dehydrated. Absolute ethanol was gradually substituted with hexamethyldisilazane (Sigma-Aldrich). Samples were left to dry, mounted on stabs with carbon adhesive discs, gold-sputtered using a Bal-Tec SCD 050 Sputter Coater (Bal-Tec AG, Balzers, Liechtenstein), and examined with a scanning electron microscope (LEO1430).

## Data Availability

The datasets used during the current study are available from the corresponding authors on reasonable request. Supplementary material is available online [75].
